# Anti-cancer Therapies Employing IL-2 Cytokine Tumor Targeting: Contribution of Innate, Adaptive and Immunosuppressive Cells in the Anti-tumor Efficacy

**DOI:** 10.3389/fimmu.2018.02905

**Published:** 2018-12-18

**Authors:** Lorenzo Mortara, Enrica Balza, Antonino Bruno, Alessandro Poggi, Paola Orecchia, Barbara Carnemolla

**Affiliations:** ^1^Immunology and General Pathology Laboratory, Department of Biotechnology and Life Sciences, University of Insubria, Varese, Italy; ^2^UOC Cell Biology, IRCCS Ospedale Policlinico San Martino, Genoa, Italy; ^3^Vascular Biology and Angiogenesis Laboratory, Scientific and Technologic Park, IRCCS MultiMedica, Milan, Italy; ^4^UOSD Molecular Oncology and Angiogenesis Unit, IRCCS Ospedale Policlinico San Martino, Genoa, Italy; ^5^UOC Immunology Unit, IRCCS Ospedale Policlinico San Martino, Genoa, Italy

**Keywords:** anti-tumor therapy, IL-2, targeting immunotherapy, chemotherapy, T-cell responses, NK cells

## Abstract

Antibody-cytokine fusion proteins (immunocytokine) exert a potent anti-cancer effect; indeed, they target the immunosuppressive tumor microenvironment (TME) due to a specific anti-tumor antibody linked to immune activating cytokines. Once bound to the target tumor, the interleukin-2 (IL-2) immunocytokines composed of either full antibody or single chain Fv conjugated to IL-2 can promote the *in situ* recruitment and activation of natural killer (NK) cells and cytotoxic CD8^+^ T lymphocytes (CTL). This recruitment induces a TME switch toward a classical T helper 1 (Th1) anti-tumor immune response, supported by the cross-talk between NK and dendritic cells (DC). Furthermore, some IL-2 immunocytokines have been largely shown to trigger tumor cell killing by antibody dependent cellular cytotoxicity (ADCC), through Fcγ receptors engagement. The modulation of the TME can be also achieved with immunocytokines conjugated with a mutated form of IL-2 that impairs regulatory T (Treg) cell proliferation and activity. Preclinical animal models and more recently phase I/II clinical trials have shown that IL-2 immunocytokines can avoid the severe toxicities of the systemic administration of high doses of soluble IL-2 maintaining the potent anti-tumor effect of this cytokine. Also, very promising results have been reported using IL-2 immunocytokines delivered in combination with other immunocytokines, chemo-, radio-, anti-angiogenic therapies, and blockade of immune checkpoints. Here, we summarize and discuss the most relevant reported studies with a focus on: (a) the effects of IL-2 immunocytokines on innate and adaptive anti-tumor immune cell responses as well as immunosuppressive Treg cells and (b) the approaches to circumvent IL-2-mediated severe toxic side effects.

## Introduction

Viewing the tumor microenvironment (TME) like a critical orchestrator in tumor biology has been a central paradigm shift of the cancer field during the past two decades. Within this time, the distinct role of tissue-residing cells in promoting or suppressing tumor growth, metastasis and resistance to therapy has been gradually elucidated. Among the host-dependent biological features of the tumor, the hallmarks defined by Hanahan and Weinberg, such as the “evading immune destruction”, the “tumor-promoting inflammation”, and the “immune orchestration of angiogenesis” point out the key role of the immune system in neoplastic disease (1). Therefore, diverse cells both from innate or adaptive immunity, as a consequence of their plasticity, have been reported to acquire an altered phenotype and functions upon TME interaction; indeed, the cross-talk between TME and immune system leads to (a) attenuation of targeting and killing of tumor cells, (b) generation of tolerogenic/immunosuppressive behavior, and (c) acquisition of pro-angiogenic activities (1–5). As soon as the immune checkpoint inhibitors entered the clinic showing important and long lasting responses, the immune system gained greater attention in cancer biology.

Cytokines are molecular messengers, allowing immune cells to communicate with each other and with the TME compartments. Growing interest has been focused in exploiting the immune system to eradicate cancer using different cytokines. Nonetheless, toxicity and dual ambivalent activities of some cytokines (tumor promoting vs. tumor inhibiting) still remain relevant issues. In this context, immunotherapy approaches, and cytokine therapy, has been a promising strategy for the treatment of cancer (6).

IL-2 cytokine displays multiple immunological effects and acts by binding to the IL-2 receptor (IL-2R). The association of IL-2Rα (CD25), IL-2Rβ (CD122), and IL-2Rγ (CD132) subunits results in the trimeric high affinity IL-2Rα*βγ*. CD25 confers high affinity binding to IL-2, whereas the β and γ subunits (expressed on natural killer (NK) cells, monocytes, macrophages and resting CD4^+^ and CD8^+^ T cells) mediate signal transduction (7, 8). It appears that the expression of CD25 is essential for the expansion of immunosuppressive regulatory T cells (Treg); on the other hand, cytolytic CD8^+^ T and NK cells can proliferate and kill target cells responding to IL-2 by the IL-2Rβγ engagement in the absence of CD25 (9). The IL-2 cytokine acts as a master activation factor for helper/regulatory T cell and NK cell proliferation, differentiation and acts as a relevant mediator for pro- and anti-inflammatory immune responses (10). Treatment with IL-2 has been associated with stable and curative regressions in patients with metastatic melanoma, renal cancer and advanced non-Hodgkin's lymphomas, representing the first effective immunotherapeutic agent (11). Generally, IL-2 can evoke some mild common side effects, such as a flu-like syndrome, fever, asthenia, nausea, and vomiting. These side effects are more frequent and more relevant when IL-2 administration is associated with chemotherapy. However, the very rare observation of potential life-threatening clinical toxicities, such as vascular leakage syndrome (VLS), severe flu-like symptoms and coma has recommended that the IL-2 be employed under the supervision of the oncologist in a hospital setting. IL-2 immunocytokines have been developed both to avoid these undesired clinical side effects, and to target cancer cells with specific anti-tumor antibodies fused with IL-2 that can elicit a potent anti-tumor immunological response within the immunosuppressive TME.

Here, according to the literature reviewing, we summarized and discussed: (a) the effects of IL-2 immunocytokines on innate and adaptive anti-tumor immune response and (b) their use in combination with other immunocytokines, chemio- and radio-therapy, immune checkpoint blockade, and immunotherapies.

## IL-2-Targeted Anti-Cancer Therapies

IL-2 was identified in 1976 as a T cell growth factor and later approved for treatment of patients with metastatic melanoma and renal cell carcinoma with beneficial results in a subset of patients (11, 12). Administration of high-dose IL-2 can be associated with relevant adverse effects that include the VLS, fever, chills, malaise, hypotension, organ dysfunction and cytopenia, well-reviewed previously (13–17). Low-doses of IL-2 lead to the preferential expansion of Treg cells; this is an unwanted effect in anti-cancer immunotherapy (18). To overcome the toxicity related to the systemic administration of IL-2 at high-dose, diverse IL-2 immunocytokines composed of IL-2 fused to antibodies directed against tumor-associated antigens (TAAs) have been tested in preclinical models with promising results (10, 19–48). Indeed, some IL-2 immunocytokines are currently in phases I–II of several clinical trials, in combination with other therapeutics (49–56) (https://www.clinicaltrials.gov). These immunocytokines showed beneficial effects for a wide range of tumor types with manageable and reversible side effects and toxicities (49–51, 53, 54, 57–60). IL-2 immunocytokines-targeted proteins are represented by either surface membrane TAAs, such as disialoganglioside 2 (GD2), epithelial cell adhesion molecule (EpCAM), carcinoembryonic antigen (CEA), CD20, and CD30 or proteins belonging to tumor extracellular matrix (ECM) like extra domain A (ED-A) and B (ED-B) of Fibronectin A-FN and B-FN, respectively and tenascin-C. The targeting of proteins expressed on the cell surface of tumor cells presents some limitations, such as the transitory expression of TAAs, the rapid immunocytokine internalization, localization and degradation into the lysosomal compartment, determining failure of the expected therapeutic effect. The tumor ECM proteins have been proposed as good targets due to the over-expression of isoforms absent or barely expressed in the ECM of normal tissues (61, 62). An overview of IL-2-immunocytokines in preclinical and clinical development for treatment of cancer is summarized in Table 1 and some examples will be discussed here.

**Table 1:** IL-2-immunocytokines in preclinical and clinical development for treatment of various types of cancer.

**IL-2-immunocytokine(target)**	**Type of study**	**Indication**	**Administration**	**Results**	**References**
L19-IL2 (B-FN)	Clinical (PhI/II)	Met. Melanoma (stage III)	IT	A complete response was achieved in 25% of patients with met. melanoma.	(49)
	Clinical (PhI/II)	Solid tumors and metastatic renal cell carcinoma	IV infusion	The immunocytokine can be safely administered in patients with advanced solid tumors with clinical activity in patients with mRCC.	(50)
L19-IL2	Preclinical	Teratocarcinoma; Orthotopic pancreatic cancer	IV	Tumor volumes were significantly reduced after therapy.	(10, 19)
L19-IL2 plus L19-TNF	Clinical (PhI/II)	Metastatic Melanoma (stage IIIC/IVM1a)	IT	Effective methods for local control of inoperable lesions.	(58)
L19-IL2 plus Dacarbazine	Clinical (PhI/II)	Metastatic Melanoma	IV infusion	More than 60% of patients were still alive 12 months of the start treatment.	(51)
L19-IL2 plus L19-TNF	Preclinical	Fibrosarcoma	IT	Totally eradication of fibrosarcoma and acquired protective immunity (100%).	(21)
	Preclinical	Teratocarcinoma		Totally eradication of smaller teratocarcinoma.	(22)
	Preclinical	Neuroblastoma		Neuroblastoma eradication in 70% of treated mice that aquired a protective immune response.	(23)
	Preclinical	Myeloma	IV	Tumor eradication in 58% of treated mice.	(24)
L19-IL2 plus RT	Preclinical	Colon, Lung and mammary carcinomas	IV	The combination therapy cured 75% of colon carcinoma bearing mice, induced addittive effect for lung carcinoma and no effect in the mammary model.	(25)
	Preclinical	Colon carcinoma	IV	Long-lasting immunological protecting against tumors in colon carcinoma models.	(26, 27)
	Preclinical	Teratocarcinoma	IV	Survival increase when RT was administrated before L19-IL2 in teratocarcinoma model.	(28)
L19-IL2 plus CTLA-4 (mouse analog of Ipilimumab)	Preclinical	Teratocarcinoma	IV/IT	The treatment induced 20–40% survival in teratocacinoma bearing mice.	(22)
	Preclinical	Colon carcinoma	IV/IT	Totally colon carcinoma eradication and acquired protective immunity (100%).	(22)
L19-IL2 plus OC-46F2 (scFv anti CD138)	Preclinical	Metastatic melanoma	IV	Combined therapy led to a complete tumor eradication until day 90 from tumor implantation in 71% of treated mice with significant differences compared to monotherapy.	(29)
L19-IL2 plus Rituximab	Preclinical	Non Hodgkin's lymphoma	IV	Complete remission in 75% or in 28.6% of the treated mice in localized or disseminated MCL, respectively.	(30)
				Complete remission in 80% or in 100% of the treated mice in two different lymphoma models.	(31)
L19-IL2 plus AAZ+-ValCit-MMAE	Preclinical	Renal cell and colon carcinoma	IV	The combination of the two agents induced complete remissions in all mice.	(63)
F8-IL2 (EDA-FN)	Preclinical	Lung adenocarcinoma	IV	Dose dependent therapeutic efficacy and survival increase in the treated mice.	(32)
F8-IL2 plus F8-TNF	Preclinical	Teratocarcinoma, Fibrosarcoma, Lung carcinoma; Melanoma	IT	Eradication of neoplastic lesions (50–75%).	(33)
F8-IL2 plus Paclitaxel or Dacarbazine	Preclinical	Melanoma	IV	Tumor eradication in the 82% of F8-IL2 plus Paclitaxel treated mice.	(21, 34)
F8-IL2 plus Sunitinib	Preclinical	Human renal cell carcinoma	IV	Partially tumor eradication (28%).	(35)
F8-IL2 plus F8-TNF (EDA FN)	Preclinical	Acute myeloid leukemia	IT	No tumor eradication.	(33)
F8-IL2 plus F8-SS-CH2CEM	Preclinical	Acute myeloid leukemia	IV	Complete and long-lasting tumor eradication in 80% of treated mice that acquired protective immunity (100%).	(36)
F8-IL2 plus Cytarabine	Preclinical	Acute myeloid leukemia	IV	Complete and long-lasting tumor eradication in 100% of immunocompetent treated mice.	(37)
F16-IL2 (TNC) plus Temozolomide	Preclinical	Human glioblastoma	IV; IP	Totally tumor eradication.	(38)
F16-IL2 plus Doxorubicin or Paclitaxel	Preclinical	Human breast cancer	IV	Significant therapeutic benefit compared monotherapies.	(39)
F16-IL2 plus Cytarabine (low dose)	Clinical (PhI/II)	Acute myeloid leukemia in patients relapsed after chemotherapies	IV infusion	Stimulation of effector cells at the bone marrow site.	(52)
hu14.18-IL2 (GD-2)	Clinical (PhI/II)	Metastatic Melanoma	IV infusion	One patient out fourteen had a partial response while four had stable disease.	(53)
	Clinical (PhI/II)	Neuroblastoma	IV infusion	Patients with disease evaluable with (123)I-MIBG scintigraphy and/or BM histology had a 21.7% CR.	(54)
	Clinical (PhI/II)	Cutaneous melanoma (stage IV)	IV infusion	There are no major objective tumor responses.	(55)
hu14.18-IL2	Preclinical	Neuroblastoma	IV/IT	Eradication of established bone marrow and liver metastases.	(20, 40)
hu14.18-IL2 plus anti-CTL-4 plus RT	Preclinical	Primary and metastatic melanoma	IT; IV	The triple-combination eradicated large tumors and metastasis, and improved animal survival compared with combinations of any two treatments.	(41)
hu14.18-IL2 plus IL-2	Preclinical	Neuroblastoma	IV	Prolonged tumor eradication of established tumors and acquired protective immunity.	(42)
antiCEA-IL2 (CEA)	Preclinical	Colon carcinoma	IV	Tumor volumes were significantly reduced after therapy.	(43)
antiCEA-IL2v	Preclinical	MC38-CEA and syngeneic pancreatic PanO2-CEA models	IV	Statistically significant increase in survival in both models.	(44)
anti CEA-IL2v Plus anti muPDL1 or Cetuximab or trastuzumab or imgatuzumab	Preclinical	Pancreatic ADK; lung, breast, colon and gastric cancers	IV	Preclinical results support the use of CEA-IL2v for combination immunotherapy with ADCC-competent or -enhanced antibodies of the IgG1 isotype, T cell bispecific antibodies that rely on CD8^+^ T effector cells and also with PD-L1 checkpoint blockade in immunogenic tumor.	(44)
				
huKS/IL-2 (EpCAM)	Preclinical	Melanoma and neuroblastoma	IT; IV	Significant anti tumor effect of immunocytokyne when IT administered vs. IV.	(27)
huKS/IL-2 plus Paclitaxel and Cyclophosphamide	Preclinical	Colon, lung and mammary carcinomas	IP; IV	Combined treatments resulted in enhanced anti tumor responses.	(45)
huKS/IL-2 plus Cyclophosphamide	Clinical (Ph II)	Small-cell lung cancer (SCLC)	IV infusion	The combination therapy was well-tolerated in extensive-disease SCLC, but did not show PFS and OS compared with best supportiva care.	(56)
DI-Leu16-IL2 (CD20)	Preclinical	Human lymphoma	IV	The modified anti-CD20 antibody fused IL2 retained full anti-CD20 activity but had enhanced ADCC respect to the unfused antibody or control Rituximab.	(46)
HRS3scFv-IL12-Fc-IL2 (CD30)	Preclinical	Hodgkin's lymphoma	IV	Suppressed tumor growth in immunocompetent mice compared to the control.	(48)
B3-IL2 (PDL1)	Preclinical	Orthotopic pancreatic carcinoma	IP	Reduced tumor growth (50%)	(47)
B3-IL2 plus TA99	Preclinical	Melanoma	IP	Slowed tumor growth and prolonged mice survival	(47)
NHS-IL2/IL2LT (DNA-histone complex)	Preclinical	Experimental lung and liver metastasis	IV	NHS-IL2LT retained anti tumor activity against established neuroblastoma and non–small cell lung cancer metastases in syngeneic mouse tumor models.	(59)
NHS-IL2+Cisplatin+RTX	Preclinical	Murine Lewis lung carcinoma	IP/IV	The combination of cisplatin plus radiotherapy with NHS-IL2 resulted in marked tumor reduction and delayed outgrowth that was statistically significant.	(60)

Among the IL-2 immunocytokines directed to ECM, the first and most studied was L19-IL-2. L19-IL-2 is specific for the angiogenesis-associated B-FN isoform selectively accumulated on tumor neovasculature; L19-IL-2 showed a good anti-tumor activity in preclinical models both in solid and hematological tumors (Table 1). A complete tumor eradication was reported when L19-IL-2 was administered in combination with CTLA-4 blockade in two syngeneic immunocompetent mouse models of teratocarcinoma and colon carcinoma; in the latter model, responder mice to this combination treatment were resistant to tumor re-challenge (22). Complete remissions of established localized lymphomas were induced when L19-IL-2 was co-administered with the anti-CD20 antibody rituximab (31). Recently, similar preclinical results were observed in renal cell carcinoma and colorectal cancer models when L19-IL-2 was co-administered with a small molecule-drug conjugate, capable of selective homing to tumor cells expressing surface carbonic anhydrase IX (63). Rekers et al. have recently reported that radiotherapy (RT) combined to systemic administration of L19-IL-2, resulted in a long-lasting immunological protection against tumors in mouse colon carcinoma. These authors hypothesize that the IL-2 immunocytokine and RT could elicit an immune-mediated abscopal effect with tumor regression far from the irradiated tumor field (25–28). Preclinical data have shown that a combination of L19-IL-2 and L19-TNF-α induced complete remission when administered as a single intratumoral injection in two immunocompetent mouse models of melanoma and sarcoma (21). These promising results led to multicenter phase II trials with the intralesional application of L19-IL-2 as single agent or in combination with L19-TNF-α in stage IIIB/IIIC and IVM1 melanoma patients as recently reviewed by Weide et al. (64).

Cancer immunotherapy holds promising synergistic potential when combined with chemotherapy and anti-angiogenic therapy. However, to our knowledge, the combined therapy of L19-IL-2 with anti-angiogenic drugs, such as bevacizumab, has not been reported until now. It is known the pro-angiogenic role of the cell surface proteoglycan syndecan-1 (CD138) whose ectodomain is a target of ADAM17 sheddase activity. The soluble ectodomain of syndecan-1 binds several matrix effectors (e.g., VEGF, FGF-2, or cytokines) and presents them to the corresponding cell surface receptors promoting angiogenesis and tumor growth (65, 66). We have recently reported that blocking syndecan-1 activity via the specific antibody OC-46F2 leads to an anti-tumor effect by inhibiting vascular maturation and tumor growth in experimental models of human melanoma and ovarian carcinoma (67). Furthermore, we have shown that OC-46F2, in combination with the L19-IL-2 resulted in complete inhibition of melanoma growth until day 90 from tumor implantation in 71% of treated mice with a significant increase of their tumor free survival (29).

The hu14.18-IL-2 immunocytokine containing a humanized anti-GD2 mAb linked to IL-2 was mainly studied as mono-therapy in neuroblastoma and in combination with anti-CTLA-4 plus RT in primary and metastatic melanoma. The triple-combination therapy eradicated large tumors and metastasis improving animal survival (41). A still ongoing phase II clinical trial using hu14.18-IL-2, reported stable disease in four patients and a partial response in one patient out of fourteen in metastatic melanoma (53) while not significant results were shown by the other two phase I/II trials already completed (54, 55).

Recently, it has been described as a novel class of monomeric tumor-targeted immunocytokines in which a single engineered IL-2 variant (IL-2v) with abolished CD25 binding is fused to the C-terminus of an antibody against the CEA or fibroblast activation protein-α (FAP). CEA-IL-2v and FAP-IL-2v demonstrated superior safety, pharmacokinetics and tumor targeting, while lacking preferential induction of Treg cells due to abolished CD25 binding. At the same time, these constructs showed monovalency and high-affinity tumor targeting as compared to classical IL-2 based immunocytokines. They retain the capacity to activate and expand NK and CD8^+^ effector T cells through IL-2Rγβ in the periphery and in the TME (Klein C.; 1st Immunotherapy of Cancer Conference (ITOC1) Munich, Germany, 2014) (44, 68).

Both in MC38-CEA and syngeneic pancreatic PanO2-CEA models, animals treated with CEA-IL2v monotherapy showed a statistically significant increase in median survival compared to untreated animals. Moreover, CEA-IL2v treatment resulted in a superior efficacy when administered in combination with PD-L1 checkpoint blockade or with ADCC competent antibodies, such as trastuzumab and cetuximab (44). In the syngeneic PancO2 model, similar results were recently described using FAP-IL2v associated with a CD40 agonistic and PD-L1 inhibitory checkpoint antibodies as reported by Nicolini V. et al. at AACR Annual Meeting 2018; Chicago, IL (https://www.clinicaltrials.gov, NCT02627274; NCT03386721).

Although it is not an immunocytokine, it is important to analyze the effects of OMCP-mutIL-2, a mutated form of IL-2 (mutIL-2) linked to a high-affinity NKG2D ligand (OMCP), which is directed to cytotoxic immune effector cells rather than tumor cells. This targeted therapy resulted in preferential binding to and activation of NK cells rather than Treg cells and a significant decrease of tumor growth was obtained after treatment with OMCP-mutIL-2 in mouse models of Lewis lung carcinoma (LLC) (69).

## Outstanding Relevance of Triggering NK Cell Activity in Therapeutic Effect of IL-2 Immunocytokines

**Figure 1:**
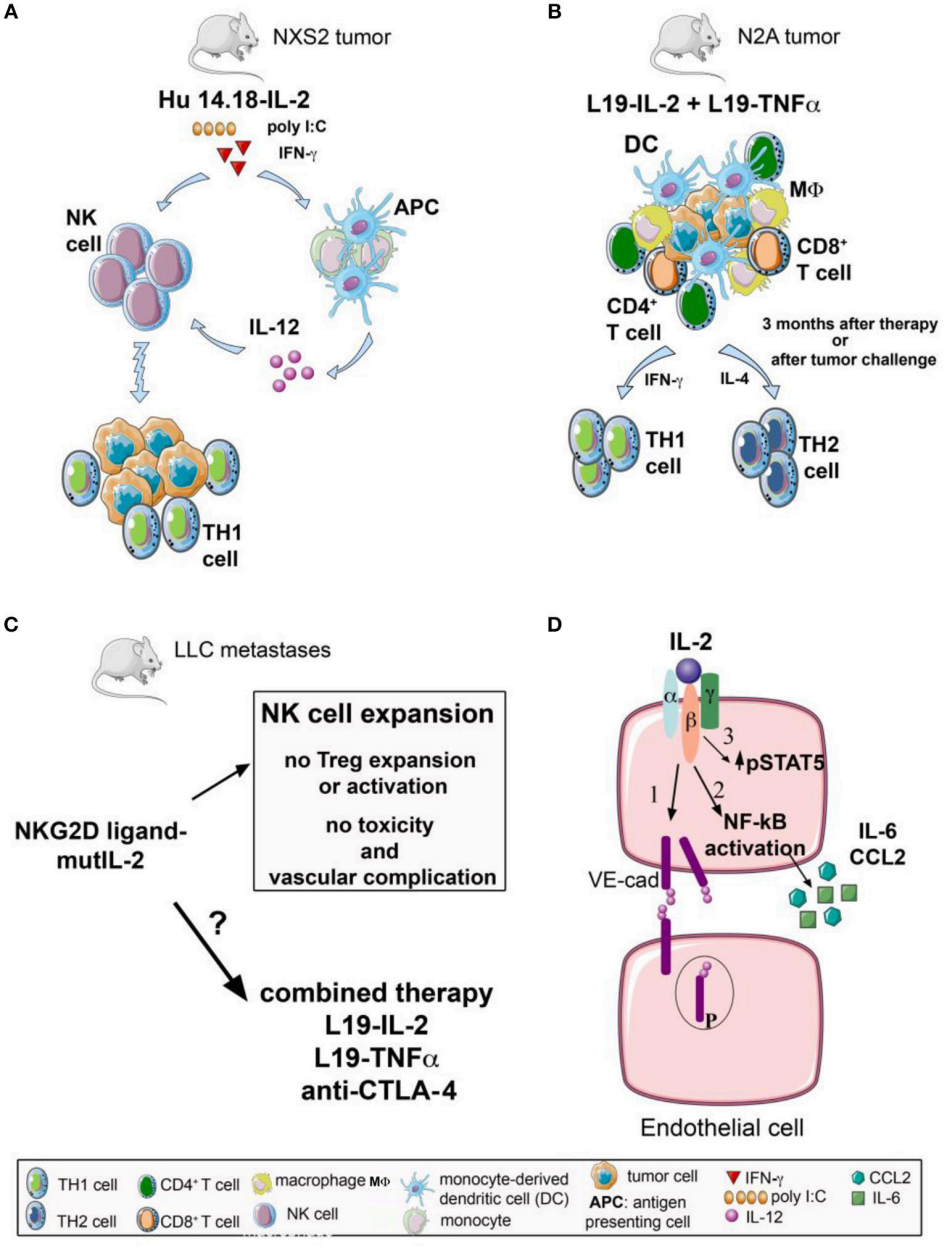
Effects on innate and adaptive immune response of IL-2 immunocytokines and IL-2 fusion protein either alone or in combination with other therapeutic approaches, and IL-2 mediated modulation of endothelial cells. **(A)** The NK cell stimulating effect of hu14.18-IL2 immunocytokine, containing a humanized anti-GD2 mAb linked to IL-2, is strongly enhanced when combined with poly I:C or recombinant mouse IFN-γ. Poly I:C and IFN-γ can be potent stimulators of antigen presenting cells (APC) as monocytes and monocyte-derived dendritic cells (mDC) that can produce IL-12, a strong inducer of NK cell cytotoxicity. This mechanism could eventually generate a Th1 microenvironment favoring anti-tumor adaptive immune response. **(B)** L19-IL-2 in combination with another immunocytokine, L19-TNF-α, shows therapeutic synergistic effects in neuroblastoma N2A murine model. 70% of systemically treated mice result in a specific long-lasting anti-tumor immune memory, with efficient priming of CD4^+^ T helper cells and CD8^+^ CTL effectors, massive tumor infiltration of CD4^+^, CD8^+^ T cells, macrophages and dendritic cells, accompanied by a mixed Th1/Th2 response. **(C)** The use of a fusion protein consisting in a mutated form of IL-2 targeting NKG2D-positive cells (OMCP-mutIL2) is employed as a monotherapy, in a preclinical model of Lewis lung carcinoma (LLC). This protocol is highly efficient in stimulating anti-tumor NK cells and their cytotoxicity with no involvement of Treg cells and in absence of vascular-related toxicity. It is still to be investigated if OMCP-mutIL2 can display a synergistic effect in those combination therapies which trigger the anti-tumor adaptive T cell response. **(D)** IL-2 is able to interact with IL-2R complex (IL-2Rβ and IL-2Rγ) on brain microvascular endothelial cells (BMEC) inducing: (1) destabilization of adherent junctions through an increase in VE-cadherin (VE-cad) phosphorylation and internalization accompanied by NF-kB activation, and (2) release of pro-inflammatory mediators, such as CCL2 and IL-6, resulting in brain oedema. Moreover, (3) IL-2 binds directly to CD25^+^ lung endothelial cells with an increase of STAT5 phosphorylation inducing pulmonary oedema.

It is well-established that IL-2 can trigger T lymphocytes to expand and acquire a therapeutic anti-tumor activity (70). Noteworthy, cytotoxic CD8^+^ T cells can be activated with IL-2 mainly by IL-2Rβγ, whereas regulatory T cells can efficiently respond to IL-2 through the IL-2Rα*βγ* complex (71–75). These peculiar features of CD8^+^ T cells have been used to design unique IL-2 molecules and favor the expansion of cytotoxic anti-tumor rather than regulatory T lymphocytes (72–75). Likewise, NK cells can respond efficiently to IL-2 through the IL-2Rβγ in the absence of IL-2Rα*βγ* heterotrimer (18, 70, 71, 76). Since NK cell can kill their target without prior sensitization or priming, they may represent a good candidate to respond to *in vivo* during administration of immunocytokines composed of IL-2 (20, 38, 70, 77). This is the case for the hu14.18-IL-2 immunocytokine, where depletion of NK cells resulted in the abrogation of the anti-tumor response detected *in vivo* in preclinical murine model of NXS2 neuroblastoma (20). Furthermore, the effect of hu14.18-IL-2 immunocytokine was strongly enhanced when combined with poly I:C or recombinant mouse IFN-γ which can be considered potent NK cell stimulating factors (20). Impressively, only NK cells, but not CD8^+^ T cells, isolated from these mice exerted a detectable cytolytic activity against the NK cell target YAC-1. This would indicate that in this murine model system NK cells can cure from neuroblastoma. It is not clear whether this effect is dependent only on IL-2-mediated activation of NK cells, or other cytolytic effector cells, such as NK-like T and/or γδ T cells not expressing CD8. In addition, both poly I:C and IFN-γ can be potent stimulators of antigen presenting cells (APC) as monocytes and monocyte-derived dendritic cells (mDC) (20, 78, 79). More importantly, APC can produce IL-12 (79), a strong inducer of NK cell cytotoxicity, and it is still to be defined whether poly I:C and IFN-γ can exert both direct and indirect effect on NK cell activation. We can speculate that the crosstalk between NK and DC, further reinforced by the triggering with poly I:C and IFN-γ of both NK and DC, could generate a positive loop to produce high IL-12 and amplify NK cell response (80, 81); this could eventually generate a Th1 microenvironment favoring anti-tumor adaptive immune response (Figure 1A).

Regarding the therapeutic efficiency of NK cells under administration of IL-2 immunocytokines, it is relevant to analyze the role of major histocompatibility complex antigens (82). It is well-established that NK cells in allogeneic hematopoietic stem cell transplant (HSCT) can show the so called “killer immunoglobulin-like receptor (KIR)/KIR-ligand incompatibility”. An improvement of leukemia control is related to a difference in HLA-I between the donor and recipient because the corresponding KIR expressed on NK cell does not recognize the HLA-I antigen (83). Thus, due to the KIR/KIR-ligand mismatch the KIR on NK cell donor does not deliver a signal in NK cell leading to inhibition of NK-cell mediated killing of residual leukemia cells present in the recipient. Importantly, the KIR/KIR-ligand mismatch can happen also in an autologous setting (84, 85). In relapsed/refractory neuroblastoma patients, hu14.18-IL-2 immunocytokine administered to the cohort with the KIR/KIR-ligand mismatch showed a better anti-tumor response to that of the matched cohort patients (82). Thus, the KIR/KIR-ligand mismatch analysis should be associated with the immunocytokine therapy to further improve the NK cell response in anti-neuroblastoma activity (82). The involvement of NK cells in the therapeutic effect of IL-2 immunocytokine has been further confirmed in targeting the tumor stroma with the F16-IL-2 immunocytokine (52). In point of fact, F16-IL-2 treatment of acute myeloid leukemia (AML) relapsed patients after HSCT led to a massive accumulation of lymphocytes in the bone marrow and CD56^+^CD16^+^ NK cells represented the most prominent increment, besides γδT and CD8^+^ T cells (52). In addition, lymphocytes appeared in contact with clusters of leukemic blasts suggesting that a recognition of tumor cells and formation of immunological synapses have been clearly established in F16-IL-2 treated patients.

## Role of Adaptive T-cell Responses in the Anti-tumor Therapeutic Efficacy in IL-2 Immunocytokines Treatments

L19-IL-2 has been largely studied in different tumor preclinical mouse models, indicating a powerful action of this compound in the ability to induce a pro-inflammatory reaction and a tumor influx of lymphocytes together with an IFN-γ response and NK and/or T cell responses. Interestingly, in the CT26 colon carcinoma murine model, L19-IL2 as well as anti-CTLA-4 mAb treatments have been shown to be active as single agents; importantly, the combination of the IL-2 immunocytokine and the immune checkpoint blocker determined an enhanced anti-tumor therapeutic effect and a prolonged survival (22). In this tumor model, treated and cured mice have been protected following tumor re-challenging, indicating memory of anti-tumor immunity. But there was no synergy between L19-IL-2 and anti-PD-1 checkpoint blockade with increased survival of CT26-tumor bearing mice; this would suggest that to obtain with this IL-2 immunocytokine a synergistic anti-tumor effect a distinctive immune checkpoint blocker should be targeted. Intratumoral L19-IL-2 in combination with L19-TNF-α immunocytokine led to a complete cure of 100% treated F9 teratocarcinoma-bearing mice. By contrast, in athymic mice, tumor rejection capacity elicited by the combination of these immunocytokines was impaired; only a delayed tumor growth in comparison to the control immunocompetent mice was observed; this points out the relevance of T cell response for the complete eradication of tumors (22). Synergy between L19-IL-2 and L19-TNF-α has been also documented in neuroblastoma models (Figure 1B) (23). L19-TNF-α is also considered a crucial element in combined anti-tumor immunotherapeutic strategies, showing encouraging results in both preclinical (86–88) and clinical studies (58).

Intratumoral injections with hu14.18-IL-2 in neuroblastoma NXS2 murine model showed an enhanced inhibition of tumor growth and prolonged survival compared with controls; this therapeutic effect involved both NK and T cells localized *in situ* and peripheral blood (40). Interestingly, after intratumoral injection, an enhanced proportion of both NK and T cells expressing NKG2A/C/E antigens in comparison to control mice and intravenous-treated tumor-bearing mice was detected. Moreover, this therapeutic approach induced a remarkable tumor infiltration of CD8^+^ CTLs, CD4^+^ T cells as well as macrophages. In *in vivo* immune cell subsets depletion assays demonstrated a key role for CD4^+^, CD8^+^ T and NK cells. Remarkably, the hu14.18-IL-2 immunocytokine has been shown to synergize with local RT and systemic checkpoint blockade (anti-CTLA-4 mAb) to eradicate large tumor and metastases in different tumor murine models (38). An important issue derived from these studies was that the cooperative effect was mediated, at least in part, by NK cells through ADCC and that the use of tumor-specific IL-2 immunocytokine led to memory T-cell responses (38).

The F8-IL-2 immunocytokine when used as monotherapy in a metastatic adenocarcinoma lung mouse model resulted in strong tumor infiltration of both CD3^+^ T and NK cells but not of Treg cells and F4/80^+^CD11b^+^ macrophages. Of note, in this model, TILs also contained an enhanced percentage of intratumoral proliferating Ki67^+^ Granzyme B^+^ CD8^+^ T cells (32).

Finally, monotherapy with OMCP-mutIL2, demonstrated a strong NK cell-mediated anti-tumor effect but no involvement of adaptive immune response, at least in the LLC model (66). It is still to be elucidated if OMCP-mutIL2 could have additive or synergistic anti-tumor effects in association with therapies that can trigger T-cell responses (Figure 1C).

## Could IL-2 be Directly Involved in the Tumor Vessels Destruction?

As mentioned above, one of the major and potentially fatal side effects upon administration of high-dose IL-2 is the VLS. This syndrome is characterized by the accumulation of fluid in the extravascular space in multiple organs, such as heart, lung, kidney, and brain. IL-2 can induce VLS acting either indirectly or directly on endothelial cells. Indeed, VLS is caused by the release of pro-inflammatory cytokines, such as TNF-α from IL-2–activated NK cells (89); in turn, this TNF-α alters the vascular permeability. Furthermore, IL-2 is able to induce both pulmonary and brain oedema binding directly CD25 expressed on lung and brain microvascular endothelial cells (BMEC); this engagement leads to disruption of the integrity of lung vascular permeability and blood-brain barrier (BBB) (90, 91).

Moreover, IL-2 interacts on BMEC with intermediate affinity IL-2Rβγ complex inducing destabilization of adherent junctions through an increase in VE-cadherin phosphorylation and internalization accompanied by NF-kB activation; this results in the release of pro-inflammatory mediators, such as CCL2 and IL-6 (Figure 1D) (91, 92). Thus, it is essential that the targeted therapy with IL-2 immunocytokines should avoid, or at least reduce, the VLS in healthy organs. Importantly, recent results from tumor-targeting IL-2 immunocytokines composed of variant forms of IL-2 lacking vascular effects and low or absent Treg cell stimulation have shown promising new avenues for IL-2 applications (44, 69, 90, 93). In addition, the molecular and biochemical mechanisms of IL-2-mediated activation of endothelial cells could be investigated *in vitro* and *in vivo* on several types of tumor-associated cells (e.g., tumor-associated endothelial cell and tumor-associated fibroblast) expressing the different chains of IL-2R; this could be potentially exploited for the treatment of tumors.

## Conclusion

IL-2 therapy can lead to durable responses in cancer patients but it is associated with significant toxicity and even life-threatening syndromes. IL-2 immunocytokines, alone or in combination with other immunocytokines, checkpoint blockade, chemio-, radio- and/or immunotherapies showed cooperative anti-tumor effects without relevant toxicities; indeed, the vast majority of preclinical tumor models have shown a strong therapeutic response to IL-2 immunocytokine. This is the firm starting point to employ IL-2 immunocytokine to improve the patients' survival and to treat metastatic cancers as well.

## Author Contributions

LM, EB, AB, AP, PO, and BC planned, organized, wrote and revised the manuscript, and prepared the figure and table.

### Conflict of Interest Statement

The authors declare that the research was conducted in the absence of any commercial or financial relationships that could be construed as a potential conflict of interest.
